# Inter-observer agreement improves with PERCIST 1.0 as opposed to qualitative evaluation in non-small cell lung cancer patients evaluated with F-18-FDG PET/CT early in the course of chemo-radiotherapy

**DOI:** 10.1186/s13550-016-0223-6

**Published:** 2016-09-22

**Authors:** Joan Fledelius, Azza Khalil, Karin Hjorthaug, Jørgen Frøkiær

**Affiliations:** 1 Department of Nuclear Medicine, Herning Regional Hospital, 7400 Herning, Denmark; 2 Department of Oncology, Aarhus University Hospital, Aarhus, Denmark; 3 Department of Nuclear Medicine & PET-Centre, Aarhus University Hospital, Aarhus, Denmark

**Keywords:** Inter-observer agreement, F-18-FDG PET/CT, Lung cancer, PERCIST 1.0, Response evaluation

## Abstract

**Background:**

The purpose of this study is to determine whether a qualitative approach or a semi-quantitative approach provides the most robust method for early response evaluation with 2′-deoxy-2′-[^18^F]fluoro-d-glucose (F-18-FDG) positron emission tomography combined with whole body computed tomography (PET/CT) in non-small cell lung cancer (NSCLC).

In this study eight Nuclear Medicine consultants analyzed F-18-FDG PET/CT scans from 35 patients with locally advanced NSCLC. Scans were performed at baseline and after 2 cycles of chemotherapy. Each observer used two different methods for evaluation: (1) PET response criteria in solid tumors (PERCIST) 1.0 and (2) a qualitative approach. Both methods allocate patients into one of four response categories (complete and partial metabolic response (CMR and PMR) and stable and progressive metabolic disease (SMD and PMD)). The inter-observer agreement was evaluated using Fleiss’ kappa for multiple raters, Cohens kappa for comparison of the two methods, and intraclass correlation coefficients (ICC) for comparison of lean body mass corrected standardized uptake value (SUL) peak measurements.

**Results:**

The agreement between observers when determining the percentage change in SULpeak was “almost perfect”, with ICC = 0.959. There was a strong agreement among observers allocating patients to the different response categories with a Fleiss kappa of 0.76 (0.71–0.81). In 22 of the 35 patients, complete agreement was observed with PERCIST 1.0. The agreement was lower when using the qualitative method, moderate, having a Fleiss kappa of 0.60 (0.55–0.64). Complete agreement was achieved in only 10 of the 35 patients. The difference between the two methods was statistically significant (*p* < 0.005) (chi-squared).

Comparing the two methods for each individual observer showed Cohen’s kappa values ranging from 0.64 to 0.79, translating into a strong agreement between the two methods.

**Conclusions:**

PERCIST 1.0 provides a higher overall agreement between observers than the qualitative approach in categorizing early treatment response in NSCLC patients. The inter-observer agreement is in fact strong when using PERCIST 1.0 even when the level of instruction is purposely kept to a minimum in order to mimic the everyday situation. The variability is largely owing to the subjective elements of the method.

**Electronic supplementary material:**

The online version of this article (doi:10.1186/s13550-016-0223-6) contains supplementary material, which is available to authorized users.

## Background

A vast palette of new types of anti-cancer agents, including more or less specific targeted treatments, has become available to many cancer patients including those with non-small cell lung cancer (NSCLC).

Unfortunately, not all patients respond to all treatments, and the matter of selecting the optimal treatment for each patient is a key element in minimizing the number of fruitless treatments with unnecessary and harmful side effects, as well as improving survival. In addition, an optimal treatment selection will contribute to keeping exploding health costs down—a pending problem, especially in medical oncology.

Early information on treatment effectiveness will be of great importance in personalized treatment planning. Consequently, this accentuates the urgency of identifying a robust method for early response evaluation. In the past, we have relied on measuring changes in tumor size, initially from chest X-rays and presently in CT images, following various classifications and recommendations: The World Health Organization (WHO) classification from 1979 [1], followed by Response Evaluation Criteria In Solid Tumors (RECIST) from 2000 [2] updated in 2009 to RECIST 1.1 [3]. Two major issues are of great importance when using these measurements: (1) tumor shrinkage takes time; a major limitation for early response evaluation using CT and (2) accurate change in size measurements can be very observer dependent. Although some studies have shown good inter-observer agreement, especially when evaluating metastasis [4–6], others (mostly primary tumor studies) have demonstrated rather poor agreement with a resulting high risk of misclassification [7–9]. In clinical trials, it is possible to overcome this observer dependency by limiting the number of radiologists performing the measurements, but this introduces a problem when comparing studies, and especially when interpreting patient data outside of the clinical trial setting.

Positron emission tomography (PET) using the tracer 2′-deoxy-2′-[^18^F]fluoro-d-glucose (F-18-FDG) combined with whole body computed tomography (F-18-FDG PET/CT), is already a well-established method for routine staging of NSCLC patients [10, 11]. Parallel to the increase in available anti-cancer pharmaceuticals, PET/CT scanners are now available in most centers treating NSCLC patients.

In spite of the lack of agreement on which method to use for quantifying the change in FDG-uptake, many studies over the past 10–15 years agree that a change in uptake during treatment contains valuable information regarding whether a patient will respond favorably to a given treatment. The metabolic response, measured as an early change in FDG-uptake, has been shown to predict both the histological response [12–18] and the post treatment evaluated response [19–21] to both chemo- and radiotherapy [22] in NSCLC patients.

In the principle, there are two different approaches to evaluate a change in FDG-uptake in non-dynamic, standard protocol F-18-FDG PET/CT scans: qualitative evaluation, which visually graduates the change as suggested by Hicks et al. [10], and a semi-quantitative approach calculating the percentage change in standardized uptake value (SUV). In 2009, Wahl et al. [23] published their suggestion for PET Response Criteria In Solid Tumors: PERCIST 1.0, thereby taking up the challenge on lack of uniformity.

This study aims to evaluate the inter-observer agreement among F-18-FDG PET/CT evaluators at our institution, using both PERCIST 1.0 response evaluation criteria [23] and the qualitative method of visual evaluation as defined by Hicks et al. [10]. Furthermore, we explore whether using the semi-quantitative method of PERCIST 1.0, as opposed to the more subjective qualitative method, will improve the agreement among observers.

## Methods

### Patients

The F-18-FDG PET/CT scans of the first 35 consecutive patients with pathologically proven stage IIB-IIIB (American Joint Committee on Cancer Staging), in-operable NSCLC enrolled in a national phase II trial were evaluated. The Danish National Ethical Board approved the trial (S209-0012). Patients were included and treated between May 2009 and March 2012 at one of two centers: Aarhus University Hospital and Odense University hospital. Written informed consent was obtained from all patients.

Induction chemotherapy consisted of Carboplatin (given as intravenous perfusion day 1) combined with Vinorelbine (60 mg/m^2^ days 1 and 8 as tablets delivered as 21-day cycles).

### Imaging

All patients had an F-18-FDG PET/CT scan performed at diagnosis (baseline) and after 2 cycles of induction chemotherapy (follow-up), prior to the radiotherapy course. The baseline F-18-FDG PET/CT scan and the follow-up F-18-FDG PET/CT scan were performed on the same type of scanner, at the same center. Nineteen of the 35 patient scans were performed at the PET-Centre, Aarhus University Hospital, using one of three integrated PET/CT scanners (Siemens Biograph TruePoint 40, Siemens Healthcare GMbH, Erlangen, Germany). A low-dose CT scan (50 mA, 120 kV) was performed for attenuation correction purposes and to determine anatomical localization. Following the scan, the images were reconstructed using the system’s AW-OSEM algorithm (21 subsets and 3 iterations) in a matrix of 168 × 168 (4.07 mm/pixel) and post-filtered with a 3.0-mm FWHM Gaussian. The patients were injected intravenously with F-18-FDG (5 MBq pr. kg +/− 10 % (min. 150 MBq, max. 700 MBq) after a fasting period of at least 6 h. The scans were obtained approximately 1 h after F-18-FDG injection.

The remaining 16 patient scans were performed at the PET-Centre, Odense University Hospital, where they were performed with either a 16-slice or 64-slice hybrid PET/CT scanner (GE Discovery 690, GE Discovery VCT, GE Discovery RX, or GE discovery STE, GE Healthcare, Broendby, Denmark) with scan length including the skull to the upper thighs. Low-dose CT scans without intravenous contrast media were performed using a standardized CT protocol, reconstructed with filtered back projection and a standard GE filter with a field of view of 50 cm (slice thickness of 3.8 mm Smart mA 30–110 mA, 140 kV, noise index 25.0, 0.8 s/rotation.

Emission images were acquired in 3-dimensional mode (2.5 min per bed position). Data were reconstructed with a 70 cm field of view, matrix size 128 × 128 or 256 × 256, slice thickness of 3.75 mm using iterative ordered-subset expectation maximization. The CT scan was also used for attenuation correction using a standard, vendor-provided, filter for this purpose.

Bedside plasma glucose concentrations were measured in all patients prior to injection of F-18-FDG using the “Precision Xceed” monitor (Abbott A/S, Abbot Diabetes Care, Copenhagen, Denmark).

### Evaluation

Eight observers, with varying levels of experience in F-18-FDG-PET/CT evaluation, were asked to participate in this study. Observers A, D, and E had little experience (one junior and two specialists, but not with FDG), observers C and G both had more than 3 years of experience with a special interest in response evaluation, and observers B, F, and H had 1–2 years of experience with FDG-PET evaluation. Observers were blinded to clinical information; additional diagnostic information including CT scans at any time point and clinical outcome information. All observers received written information on the qualitative method of visual evaluation of response as defined by Hicks et al. [10], and of the PERCIST 1.0 response evaluation criteria [23].

F-18-FDG-PET/CT scans at baseline and at follow-up after 2 cycles of induction chemotherapy were evaluated; firstly, according to the qualitative method where the response categories were recorded together with any comments on difficulties (e.g., whether a new focus was suspected to be malignant or benign, atelectases, etc,). Secondly, all observers were asked to evaluate the same patients using PERCIST 1.0, including SUVpeak values corrected for lean body mass (SULpeak). All observers reported SULmean liver and SD for a standard 3 cm ROI in the right lobe at baseline. The minimum level for evaluation as defined by PERCIST 1.0 (1.5 × mean liver SUL + 2SD) was automatically calculated in the report file. All observers reported the highest observed SULpeak value in the most intense tumor lesion at baseline and at follow-up, not necessarily the same lesion; the percentage change was automatically calculated in order to rule out calculation errors. They also reported the final PERCIST response categories for each patient and comments in case of difficulties. A consensus classification was made for comparison, choosing the response category the majority of observers used, or in case of an equal split (three difficult cases in the qualitative analysis), the comments on difficulties reported by the observers were used to determine the category for consensus and confirmed by reevaluation by one experienced observer. The criteria for categorizing response by the two methods are summarized in Table 1.

**Table 1:** A summary of the definitions of measurability and response categories

	Visual evaluation	PERCIST 1.0
Measurable lesions	FDG avid above background	SUL peak at least 1.5 times SULmean liver + 2 SD
CMR*	FDG avid lesions revert to background of normal tissue in which they are located	Disappearance of all measurable lesion to background blood-pool levels
PMR*	Significant reduction in intensity of tumor metabolic activity	Reductions of min. 30 % in target SULpeak, with an absolute drop of at least 0.8. No new lesions
SMD*	No visible change in metabolic activity of tumors	Not CMR, PMR, or PMD
PMD*	Increase in intensity or extent of tumor metabolic activity or new lesions typical for cancer	More than 30 % increase in target SULpeak, with an absolute increase of at least 0.8. Or visible increase in extent without drop in SULpeak. Or appearance of new FDG avid lesions typical of cancer, not related to treatment or infection

* Abbreviations: CMR = Complete Metabolic Response, PMR = Partial Metabolic Response, SMD = Stabile Metabolic Disease and PMD = Progressive Metabolic Disease

### Statistical analysis

All statistics were calculated using www.statstodo.com. The inter-observer agreement in reporting response categories was evaluated using weighted Fleiss’ kappa for multiple raters, for both the qualitative method and PERCIST 1.0., Cohen’s kappa was used for pair-wise comparison of observers and for evaluating agreement between the two methods for each observer. All kappa values are reported as linear weighted kappa (95 % confidence interval). Kappa values were interpreted according to Landis and Koch [24] as summarized in Table 2. Differences between the two methods were tested using the chi-squared test. Intraclass correlation coefficients (ICC) were used to evaluate the correlation between different observers in the case of SULpeak and liver SUL measurements, interpreted using a similar scale as for kappa values (Table 2).

**Table 2:** Interpretation of kappa values and intraclass correlation coefficients (ICC) based on Landis and Koch [24]

Kappa value/ICC	Degree of agreement
0.00–0.20	Poor agreement
0.21–0.40	Fair agreement
0.41–0.60	Moderate agreement
0.61–0.80	Strong agreement
0.81–1.00	Near complete agreement

## Results

**Fig. 1:**
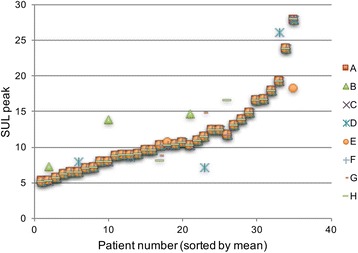
SULpeak values at baseline for all eight observers (*A–H*). *Note*: where agreement is complete all eight observations are stacked

**Fig. 2:**
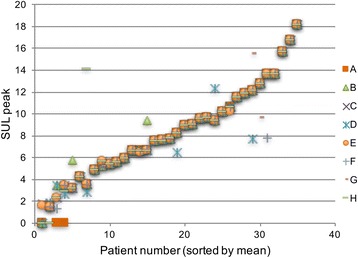
SULpeak values at follow-up for all eight observers (*A–H*). *Note*: where agreement is complete all eight observations are stacked

**Fig. 3:**
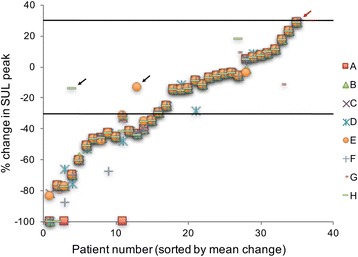
SULpeak percentage change between baseline and follow-up for all eight observers (*A–H*). *Note*: where agreement is complete all eight observations are stacked. The *horizontal lines* represent the 30 % change levels used for discrimination between response categories according to PERCIST 1.0. The *arrows* mark three patient in whom a discrepancy results in different categorization. *Red arrow*: A misclassification of possible clinical importance where *G* reported PMD (30.8 % increase in SULpeak) and the other observers reported SMD (28.6 %). The *two black arrows*: *E* reports SMD (13.6 % SULpeak change) the others reported PMR (43 % change) and an example where *H* report SMD (14.4 % decrease in SULpeak) and the other observers report PMR (69.9 % decrease)

All observers evaluated all 35 patients, assigning each patient a response category (Table 1). All observers considered all patients evaluable. Although all observers were informed to report SULpeak values for background when the response was considered complete, a SULpeak value of 0.0 was reported at follow-up in nine cases. Six of these were for a single patient showing complete response. In spite of this, the ICC for follow-up SULpeak was 0.9537, the ICC for baseline SUL peak was 0.9643, and the ICC for percentage change in SULpeak was 0.9585, all translating into almost perfect agreement, as defined in Table 2. The SULpeak values for each patient at baseline, follow-up, and percentage change in SUL peak between baseline and follow-up are presented in Figs. 1, 2, and 3.

**Fig. 4:**
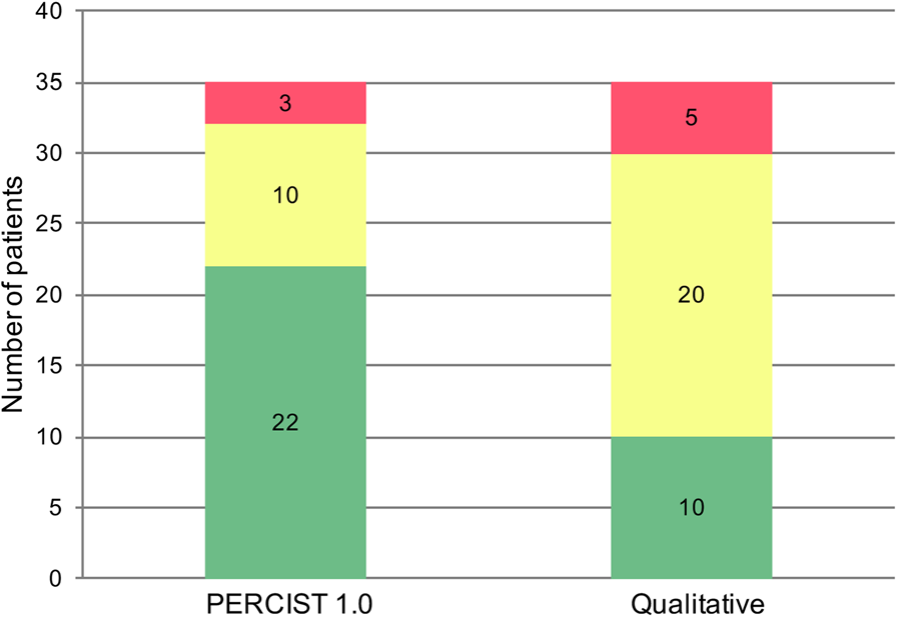
Agreement among eight observers. *Red* is “multiple level disagreement” defined as more than one response category difference, i.e., some observers reported progressive disease and some reported partial metabolic response. *Yellow* is “single level disagreement” defined as only one response category difference for one or more observers. *Green* is full agreement among all observers

**Fig. 5:**
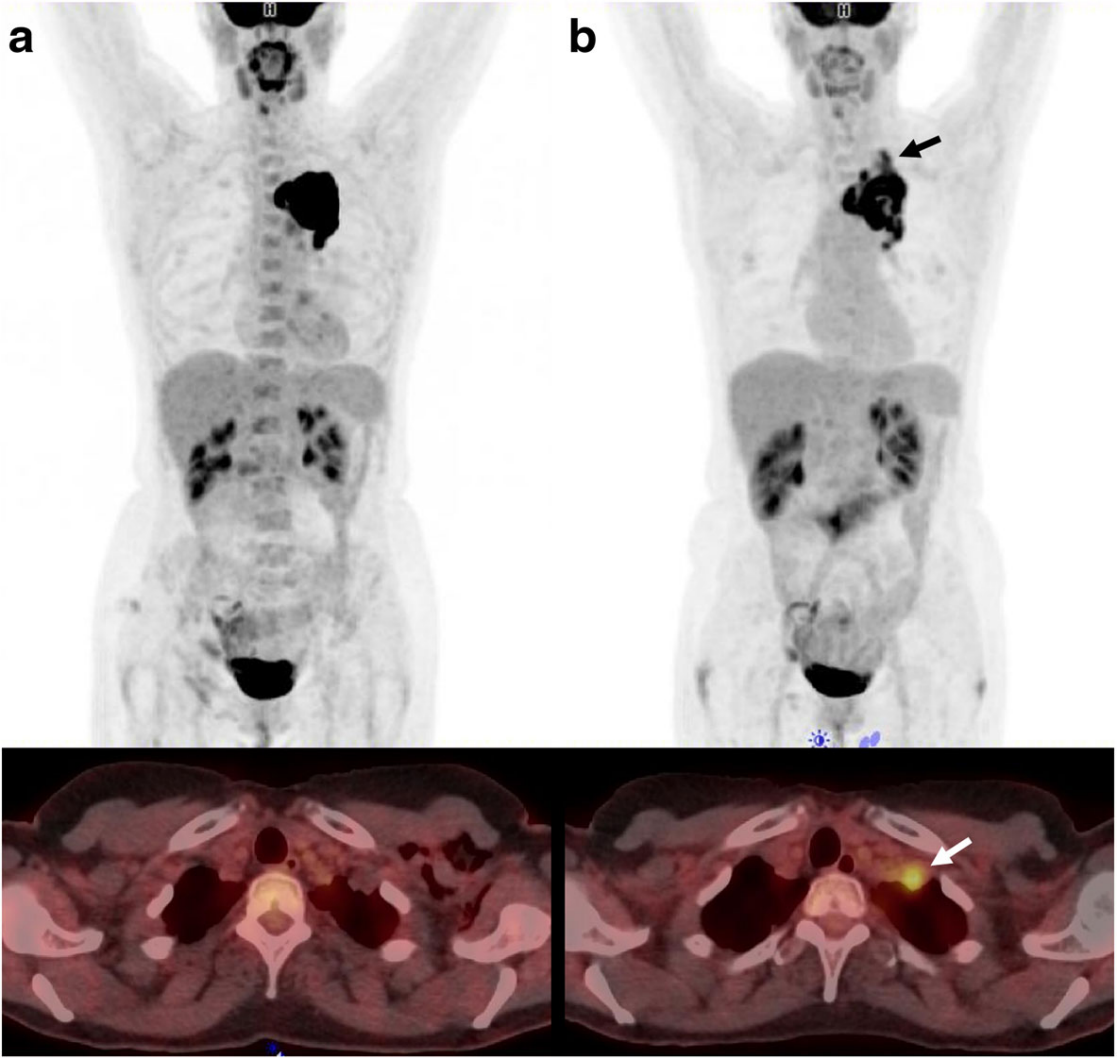
A typical case of disagreement between observers. **a** is the baseline and **b** is follow-up. On the *bottom*, one of the foci with uptake on follow-up is presented. In general, all observers found that a decrease in FDG-uptake was seen on follow-up (**b**), but three observers found that new areas with high FDG-uptake represented new malignant lesions (PMD) and five observers evaluated PMR, indicating the foci most likely to be benign. All observers reported a 51 % SULpeak decrease

Using PERCIST 1.0 for categorizing response, there was complete agreement between all 8 observers in 22 of the 35 patients; the Fleiss kappa was 0.76 (0.71–0.81) in the strong agreement category (Fig. 4). Of the 13 cases of disagreement, 10 were attributed to the subjective evaluation of complete response, new foci evaluated as malignant and visual growth of the tumor. Two of the remaining three were attributed to a numerical rounding off uncertainty and the last one to an unexplained deviating SULpeak value at baseline for one observer. When using the qualitative method, there was complete agreement among all 8 observers in only 10 of the 35 patients (statistically significant difference; *p* < 0.005), and the Fleiss kappa was significantly lower 0.60 (0.55–0.64), in the moderate agreement category. Both single level of difference (SMD/PMR or PMR/CMR) and multilevel difference in all cases SMD/PMD were lower using PERCIST as compared to visual evaluation. A comparison of the levels of agreement is presented in Fig. 5. The multilevel agreement is considered clinically relevant in all cases since progression during chemotherapy is considered to be a contraindication to continuing curatively intended chemo-radiotherapy, also single level agreement involving SMD/PMD differences is equally important. Evaluating qualitatively in 15 patients, we found these clinically important differences and in 9 patients using PERCIST. In most cases, it was only one observer deviating from the rest. A summary of these cases is presented in Table 3.

**Table 3:** A summary of cases with disagreement in response categories for qualitative and PERCIST evaluation with a possible clinical consequence

	Qualitative evaluation				PERCIST evaluation		
Case	Obs.	Categories	Cons.	Reason for PMD	Obs.	Categories	Cons.
5	1	SMD/PMD	SMD	Increased uptake	0		
6	1	SMD/PMD	SMD	Increased uptake	0		
7	1	SMD/PMD	SMD	NA	0		
8	*2*	PMR/PMD	PMR	New focus	*3*	PMR-PMD	PMR
14	*5*	PMR-PMD	PMD	New focus	*3*	PMR/PMD	PMR
16	*2*	SMD/PMD	PMD	Increased uptake	1	SMD/PMD	SMD
20	1	SMD/PMD	PMD	New focus	*3*	SMD/PMD	PMD
21	1	SMD/PMD	SMD	New focus	1	SMD/PMD	SMD
28	1	SMD/PMD	PMD	Increased size	1	SMD/PMD	SMD
30	*3*	PMR/PMD	PMR	New focus	*4*	PMR/PMD	PMR
31	1	SMD/PMD	SMD	Increased uptake	0		
32	1	PMR/PMD	PMR	New focus	0		
33	*4*	SMD/PMD	SMD	Increased uptake	0		
34	1	SMD/PMD	PMD	Increased size	1	SMD/PMD	SMD
35	*2*	SMD/PMD	PMD	New focus	*3*	SMD/PMD	PMD

Obs. is number of observers disagreeing with consensus (Cons.)

Cases with more than one observer disagreeing are considered most important

Focusing on the cases with more than one observer disagreeing, it is mainly owing to “new focus” found by some observers. An example is presented in Fig. 5.

**Fig. 6:**
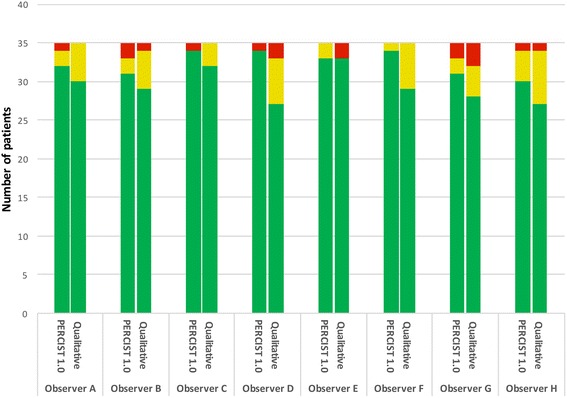
Observers agreement with the consensus categorization for qualitative end PERCIST evaluation. *Red* is “multiple level disagreement” defined as more than one response category difference between the observer and the consensus categorization. *Yellow* is “single level disagreement” defined as only one response category difference. *Green* is full agreement between observer and the consensus categorization

A comparison of observer interpretation, “one-on-one”, was also performed for each method in supplement to the multiple reader comparison. The pair-wise comparison kappa values (presented in Table 4) for PERCIST 1.0 (range 0.60–0.88) corresponded to strong agreement in 19 of the 28 compared pairs and near complete agreement for 9 compared pairs. Comparing each observer with the consensus evaluation, the kappa values (range 0.70–0.95) correspond to four observers in “near complete agreement” and four in “strong agreement” with the consensus. Interestingly, observer G (an experienced observer) had the lowest kappa values. In contrast, the qualitative evaluation of pair-wise comparison kappa values (range 0.50–0.76) corresponded to 8 pairs with moderate agreement and 20 pairs with strong agreement (presented in Table 5). Using this method, no observer-pair reached near complete agreement. Though compared with the consensus, the kappa values (range 0.66–0.90) correspond to six in strong agreement and two in near complete agreement with the consensus. The level of disagreement was not correlated to the level of experience. The observers’ agreement with “consensus” for both methods is presented in Fig. 6.

**Table 4:** Cohen’s linear weighted kappa values for pair-wise comparison of observers for PERCIST 1.0

Observer	*A*	B	C	*D*	*E*	F	G	H
B	0.74 (0.54–0.94)							
C	0.88 (0.75–1.01)	0.85 (0.67–1.02)						
*D*	0.72 (0.48–0.96)	0.69 (0.43–0.96)	0.83 (0.61–1.06)					
*E*	0.70 (0.47–0.93)	0.67 (0.42–0.93)	0.82 (0.62–1.03)	0.82 (0.62–1.03)				
F	0.83 (0.64–1.03)	0.72 (0.47–0.96)	0.85 (0.68–1.06)	0.87 (0.68–1.06)	0.86 (0.70–1.01)			
G	0.60 (0.32–0.88)	0.65 (0.40–0.90)	0.63 (0.33–0.92)	0.79 (0.59–1.00)	0.69 (0.42–0.95)	0.73 (0.48–0.99)		
H	0.63 (0.38–0.87)	0.67 (0.46–0.88)	0.64 (0.40–0.90)	0.73 (0.52–0.94)	0.71 (0.49–0.93)	0.67 (0.45–0.90)	0.77 (0.59–0.94)	
Cons.	0.80 (0.60–1.00)	0.76 (0.53–0.99)	0.92 (0.75–1.08)	0.92 (0.75–1.08)	0.91 (0.71–1.03)	0.95 (0.87–1.04)	0.70 (0.44–0.94)	0.72 (0.51–0.94)

Kappa (95 % confidence interval), Cons. is the consensus evaluation

Observers A, D, and E (in italics) had little experience, B, F, and H had 1–2 years of experience and C and G had more than 3 years of experience

**Table 5:** Cohen’s linear weighted kappa values for pair-wise comparison of observers for “qualitative” evaluation

Observer	*A*	B	C	*D*	*E*	F	G	H
B	0.57 (0.37–0.78)							
C	0.71 (0.55–0.87)	0.74 (0.55–0.92)						
*D*	0.52 (0.31–0.73)	0.66 (0.43–0.88)	0.66 (0.45–0.86)					
*E*	0.67 (0.45–0.89)	0.63 (0.39–0.87)	0.76 (0.56–0.97)	0.69 (0.48–0.89)				
F	0.68 (0.50–0.86)	0.50 (0.27–0.73)	0.64 (0.45–0.83)	0.51 (0.28–0.74)	0.67 (0.46–0.88)			
G	0.54 (0.29–0.78)	0.76 (0.56–0.97)	0.57 (0.33–0.82)	0.75 (0.58–0.93)	0.66 (0.42–0.90)	0.53 (0.29–0.78)		
H	0.65 (0.46–0.84)	0.74 (0.59–0.90)	0.68 (0.49–0.87)	0.61 (0.40–0.81)	0.64 (0.43–0.86)	0.52 (0.30–0.73)	0.64 (0.45–0.84)	
Cons.	0.80 (0.66–0.95)	0.76 (0.58–0.95)	0.90 (0.79–1.01)	0.69 (0.48–0.89)	0.86 (0.68–1.05)	0.74 (0.57–0.91)	0.66 (0.42–0.90)	0.71 (0.52–0.90)

Kappa (95 % confidence interval), Cons. is the consensus evaluation

Observers A, D, and E (in italics) had little experience, B, F, and H had 1–2 years of experience and C and G had more than 3 years of experience

There was a strong agreement for all 8 observers when comparing PERCIST 1.0 and qualitative evaluation for each observer (kappa values ranged between 0.64 and 0.79) (Table 6).

**Table 6:** Cohen’s linear weighted kappa values for comparison of the qualitative method and PERCIST 1.0

Observer	Cohen’s kappa (95 % CI)
*A*	0.79 (0.64–0.94)
B	0.79 (0.64–0.94)
C	0.71 (0.54–.089)
*D*	0.64 (0.46–0.82)
*E*	0.73 (0.55–0.91)
F	0.78 (0.61–0.96)
G	0.70 (0.52–0.88)
H	0.70 (0.53–0.87)

Observers A, D, and E (in italics) were inexperienced

The mean liver SUL was reported at baseline by all observers and showed only moderate agreement with an ICC of 0.58, and the corresponding minimum value for SULpeak for evaluation also only showed moderate agreement (ICC of 0.52),

## Discussion

The main finding of this study is that PERCIST 1.0 provides a higher overall agreement between observers than when using the qualitative approach in categorizing early treatment response in NSCLC patients with FDG-PET/CT.

There has previously been some investigation into the inter-observer variability of using F-18-FDG PET/CT for staging and recurrence evaluation in various types of cancers; some using qualitative evaluation [25–28] with generally only moderate agreement among observers, and some using SUV-based evaluation [5, 29–31] which demonstrate better (mostly near perfect) agreement among observers. To our knowledge, only two previous studies have evaluated the inter-observer variability for response evaluation; Jacene et al. [8] and Benz et al. [32] for sarcoma and lung cancer patients, respectively, both using a semi-quantitative approach and demonstrating almost perfect agreement among observers.

Since SUV has been established as a parameter with high reproducibility for pre-therapeutic evaluation in various cancer types, including NSCLC [33–36], it is important to evaluate the observer’s contribution to the overall variation.

This study was designed to provide additional information to the previous studies on inter-observer variability, especially in the response evaluation setting, and to test the hypothesis, which the more subjective, visual approach to interpretation will have a larger dependency on the individual observer than the more objective method: PERCIST 1.0. We deliberately chose to provide all evaluators with rather sparse information so as to mimic the everyday clinical situation; the aim being to evaluate actual agreement as it would present when introducing the methods into routine evaluations. Measuring SULpeak is incorporated in the PERCIST method, since it has been shown to be a more reproducible parameter than the more frequently used SUVmax [35, 37]. Furthermore, SULpeak has recently been shown to be independent of acquisition time [38], but is potentially slightly more observer dependent than SUVmax, especially in low uptake tumors.

We found that there was an almost perfect agreement among eight observers when reporting SULpeak values for all baseline values, follow-up values and percentage changes. The ICC’s for SULpeak correspond well with most other studies [8, 29–31]. We did not achieve complete agreement, which also has been demonstrated for methods using SUVmax in sarcoma patients [32] and pulmonary nodules [5] previously. This is mostly explained by the reporting of SULpeak = 0.0 when a complete response was observed at follow-up by some observers, and in some instances reporting the wrong SUV (corrected for body weight instead of LBM, as this was the software default setting).

We report a statistically significant, higher rate of total agreement among observers using PERCIST 1.0 as compared to the qualitative method. Both methods however, show a strong agreement among observers. To our knowledge no other studies have made this direct comparison. A few studies have shown, that when using qualitative, visual approaches to staging various cancers, the inter-observer agreement is moderate in most cases [25, 26, 28, 37, 39], even the well established Deauville criteria for lymphoma evaluation was shown in a study by Itti et al in 114 diffuse large B-cell lymphoma patients to have only moderate agreement among experienced observers [40].

Clearly, it is the more subjective parts of the PERCIST 1.0 that contribute most to the disagreement among evaluators. This was indicated by the almost perfect correlation between SULpeak values and by the observers’ individual added comments on new foci, tumor growth, and inclusion of atelectases for the 13 patients, where disagreement was found, all which helped highlight this disagreement. The potentially important discrepancies was mainly owing to new FDG avid foci, and whether or not they were to be considered malignant, stressing the importance of confirming the findings with biopsies.

Comparing with reported inter-observer agreement among CT measurements [7–9], the observed strong agreement when using PERCIST 1.0 would indicate that this method is in fact a helpful tool for evaluating response using F-18-FDG PET/CT; i.e., the combination of a semi-quantitative parameter with an overall visual evaluation, can provide acceptable agreement among even rather inexperienced observers. There is still room for improvement though, and when introducing this method into our daily routine, we intend to include a consensus reading between at least two evaluators. The moderate agreement seen in liver SULmean values is expected to improve with new versions of software automatically placing and defining liver VOI’s as according to PERCIST guidelines, which have been introduced as of late.

This study is limited by the lack of follow-up data. Previously, we have shown in a smaller study [41] that using PERCIST for response evaluation in a similar group of patients predicts survival after 2–4 cycles of chemotherapy. However, further studies are needed in order to evaluate which of the two approaches provides the most relevant clinical information.

## Conclusions

SUV (in this case SULpeak) is a robust parameter when considering inter-observer variability. For a large group of observers, with varying levels of experience, we have shown that the semi-quantitative approach of PERCIST 1.0 provides a significant higher overall agreement among observers than a more qualitative approach when categorizing the response in NSCLC patients early during treatment. The inter-observer agreement is strong when using PERCIST 1.0 even when the level of instruction is purposely kept to a minimum in order to mimic the everyday clinical situation; it is thus a very robust method, ready for routine use. The variability is largely owing to the subjective elements in the semi-quantitative method.

The dataset supporting the conclusions of this article is included as Additional files 1 and 2.

## Additional files

Additional file 1:
**Figure S7.** Agreement in liver SULmean at baseline among eight observers. **Figure S8.** Agreement in minimal SULpeak calculation at baseline among eight observers. (DOCX 567 kb) Additional file 2: All observers reported data. (XLSX 43 kb)
