# Supplementary material for: Inter-observer agreement improves with PERCIST 1.0 as opposed to qualitative evaluation in non-small cell lung cancer patients evaluated with F-18-FDG PET/CT early in the course of chemo-radiotherapy
Source: EJNMMI Res. 2016 Sep 22;6:71. doi: 10.1186/s13550-016-0223-6 (PMC5031580; doi:10.1186/s13550-016-0223-6)

Supplementary Figure 7: Agreement in liver SULmean at baseline among 8 observers


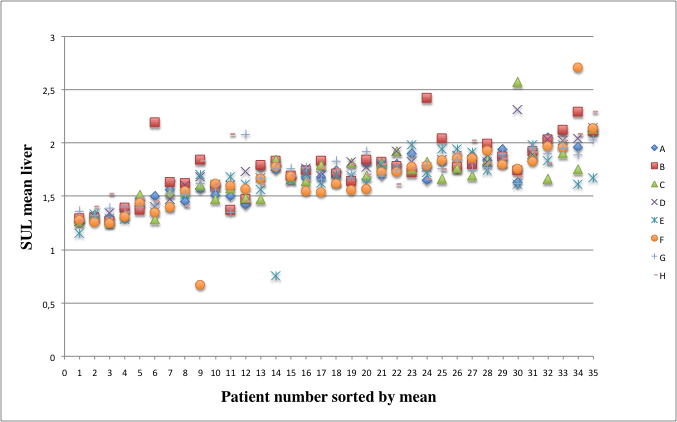


Supplementary Figure 8: Agreement in Minimal SULpeak calculation at baseline among 8 observers


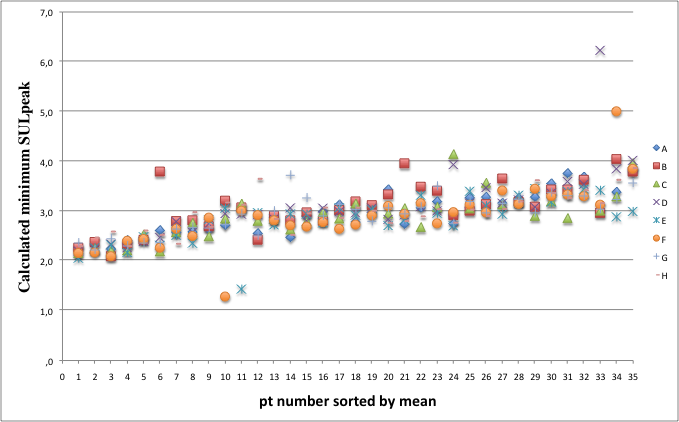

Supplement: Additional file 1: — Figure S7. Agreement in liver SULmean at baseline among eight observers. Figure S8. Agreement in minimal SULpeak calculation at baseline among eight observers. (DOCX 567 kb) [file 13550_2016_223_MOESM1_ESM.docx]
